# Neurosurgical Patient’s Quality of Life: A Questionnaire-Based Study

**DOI:** 10.7759/cureus.64553

**Published:** 2024-07-15

**Authors:** Sofia Iliopoulou, Maria Tzika, Nikolaos Foroglou

**Affiliations:** 1 Neutropenic Care Unit, AHEPA University Hospital, Thessaloniki, GRC; 2 Neurosurgery, Aristotle University of Thessaloniki, AHEPA University Hospital, Thessaloniki, GRC

**Keywords:** health care providers, neurosurgical patients, epidemiology, severity of disease, quality of life

## Abstract

Introduction: The burden of disease, as well as social and epidemiological factors, have a clear impact on a patient’s quality of life. Especially in neurosurgery, patients commonly experience a decline in their quality of life. This study aims to assess the quality of life of neurosurgical patients and evaluate the impact of epidemiologic and disease-related factors.

Methods and material: Adult, non-trauma neurosurgical patients were included in the study, which took place in the Neurosurgical Department at AHEPA University Hospital. Self-administered questionnaires including the 36-Item Short Form Survey Instrument (SF-36) and the EQ-5D-5L were used to assess the overall patient’s quality of life. Additionally, all patients were asked to provide data on the perceived severity of the disease and the extent of information regarding their health condition. Epidemiologic factors including gender, age, education level, and rural or urban living environment were also taken into account. Statistical analysis was performed to assess the impact of the aforementioned parameters on the patient’s quality of life.

Results: In total, 74 patients were included in the study (56.9% male, mean age: 51 years). In general, better mean scores were observed in general health perception, vitality, social role functioning, and mental health, whereas the lowest values were detected in the reported physical and emotional role functioning. No statistically significant differences were observed among genders. Age was found to impact the general health perception and EQ-VAS (visual analog scale) score, while physical functioning presented significant differences depending on the patient’s living environment and education level, with better scores for rural residents and secondary education graduates. The perceived severity of the health condition presented a significant negative effect on the EQ-VAS score, while it affected significantly physical functioning, with better outcomes reported by patients dealing with more serious diseases. Finally, in most of the evaluated categories, the level of information seemed to increase the reported quality of life, even though statistical significance was not confirmed.

Conclusion: Quality of life should be taken into account when treating neurosurgical patients, and utilizing measuring tools assists in objectively evaluating their well-being. Most parameters that influence the patient’s quality of life are fixed. Therefore, based on our study results, healthcare professionals should prioritize providing comprehensive information regarding the patient's disease and treatment, as the level of information seems to improve the overall patient’s quality of life.

## Introduction

Health has a multidimensional meaning that differs among individuals and is defined as a state of complete physical, mental, and social well-being. Quality of life is affected by an individual’s health status, in combination with social and personal factors, including epidemiologic characteristics. Even though health disorders play a crucial role in determining one’s quality of life, the eradication of disease by healthcare providers should be accompanied by a humanistic attempt to improve the patient’s overall well-being [1]. Especially in neurosurgical patients, a decline in the quality of life upon diagnosis and treatment of the disease is consistent, as the functional status of the patient is affected by the disease process [2,3].

Several questionnaires, which have been proposed in the literature, can assist healthcare providers in assessing the living standards and facilitating a patient’s progress evaluation and overall healthcare improvement. The 36-item Short Form Survey Instrument (SF-36) covers eight health domains, including the participant’s limitations in physical, social, and usual activities, based on physical or mental health, pain, general health, and vitality. In 2009, the EuroQol group introduced a five-level EQ-5D questionnaire that takes into account five areas: mobility, self-care, usual activities, pain or discomfort, and anxiety or depression; it also presents a visual analog scale (EQ-VAS). Both measuring tools have been standardized for the Greek population [4,5].

The aim of this study is to assess the quality of life of neurosurgical patients and evaluate the impact of epidemiologic and disease-related factors.

## Materials and methods

All adult patients hospitalized in the Neurosurgical Department (AHEPA University Hospital), during a randomly selected three-month period, were included in this study. Patients with impaired level of consciousness (Glasgow Coma Scale <15), difficulty in communicating in the Greek language, or unwillingness/inability to provide consent were excluded from the study. Patients were classified into three age groups (18-39 years old, 40-59 years old, and >60 years old) and education levels (primary school graduates and secondary school and higher education graduates), while they were also asked to report whether they lived in an urban or rural environment. Patients received two self-administered questionnaires, the SF-36, and the EQ-5D-5L, and they were additionally asked the following questions, with three-leveled answers. Question 1: “How serious do you feel your health condition is?” possible answers: “a little serious,” “quite serious,” or “very serious.” Question 2: “To what extent do you feel informed about your health condition?” possible answers: “I know some information about my condition,” “I know enough about my condition,” or “I know a lot of details about my condition.”

All data were statistically analyzed using IBM Statistics 25. Descriptive values were assessed by mean value and SD. Quantitative data were compared by performing a t-test for independent values and an ANOVA test or Mann-Whitney and Kruskal-Wallis tests, depending on the normality of the data distribution, respectively. The level of significance was set at p-value <0.05.

## Results

In total, 131 patients were hospitalized during the study period and 74 of them met the inclusion criteria. Among the participants, 42 patients were male (56.8%) and ages ranged from 18 to 79 years (mean age was 51 years). Patients younger than 40 years old were 17.6% of the total sample, 40 to 59 years old were 47.3%, and older patients (60-79 years old) were 35.1%. Patients lived in an urban environment in 66.2% of cases and the countryside in 33.8%, whereas regarding their education level, patients were primary school graduates in 13.5%, high school graduates amounted to 58.1%, and higher education graduates to 28.4%.

Regarding the patients’ self-reported answers on the seriousness of their condition, 35.1% of patients declared that their health condition is a little serious, 40.5% reported that their condition is quite serious, while the rest thought that their health issue is very serious. The majority of patients noted that they knew enough or even a lot regarding their health condition (45.9% reported that they knew enough details and 40.5% that they knew a lot), while 13.5% of patients reported that they knew only some details concerning their disease.

The SF-36 questionnaire (per category) and EQ-VAS scores are presented in Table 1, while statistical analysis is noted in Table 2.

**Table 1:** Descriptive statistics of SF-36 and EQ-VAS results (quantitative values per category) SF-36, 36-Item Short Form Survey Instrument; VAS, visual analog scale

	Mean value	SD	Minimum value	Maximum value
Physical function	58.04	22.73	15	100
Physical role	51.21	29.93	25	100
Bodily pain	23.3	22.08	10	100
General health	58.54	19.55	17	100
Vitality	50.61	19.48	5	85
Social functioning	44.9	20.37	13	100
Emotional role	65.21	29.89	33	100
Mental health	54.29	19.68	8	92
EQ-VAS	67.42	19.85	20	95

**Table 2:** Statistical analysis (p-values) of SF-36 and EQ-VAS results, based on patient’s gender, age group, living environment, reported seriousness of their health condition, and the extent of knowledge about it ^a^t-test for independent samples bMann-Whitney test ^c^ANOVA ^d^Kruskal-Wallis test (Dunn’s post hoc significant differences are noted in the results) SF-36, 36-Item Short Form Survey Instrument; VAS, visual analog scale

	Gender	Age	Living environment	Education	Seriousness of health condition	Knowledge regarding health condition
Physical function	0.39^b^	0.431^d^	0.011^b^	0.011^d^	0.015^d^	0.386^d^
Physical role	0.401^b^	0.828^d^	0.868^b^	0.834^d^	0.941^d^	0.667^d^
Bodily pain	0.726^b^	0.76^c^	0.475^a^	0.28^c^	0.887^c^	0.23^c^
General health	0.38^a^	0.012^d^	0.269^a^	0.593^d^	0.051^c^	0.055^c^
Vitality	0.868^a^	0.701^c^	0.704^a^	0.718^d^	0.061^c^	0.782^c^
Social functioning	0.788^b^	0.947^c^	0.977^a^	0.277^c^	0.498^d^	0.291^c^
Emotional role	0.829^b^	0.488^d^	0.256^b^	0.472^d^	0.413^d^	0.675^d^
Mental health	0.611^b^	0.528^d^	0.816^b^	0.975^d^	0.074^c^	0.961^d^
EQ-VAS	0.52^b^	0.09^d^	0.959^b^	0.707^d^	0.04^d^	0.058^d^

In general, better mean values were observed in general health perception, vitality, social functioning, and mental health, whereas the lowest values were detected in physical and emotional functioning. Statistically significant differences were not observed between genders. General health perception was found significantly better in patients younger than 40 years old, whereas patients between 40 and 59 years old were the most concerned ones about their general health (Kruskal-Wallis p-value=0.012, Dunn’s post-hoc analysis p-value=0.006 between groups <40 years old and 40-59 years old). The physical functioning of patients presented significant differences depending on the patient’s living environment, as well as education level. Particularly, regarding physical functioning, patients living in a rural environment reported better outcomes than patients living in urban areas (Mann Whitney p-value=0.011), whereas higher education graduates scored significantly lower than secondary education graduates (Kruskal-Wallis p-value=0.011, Dunn’s post-hoc analysis p-value=0.009).

**Figure 1:**
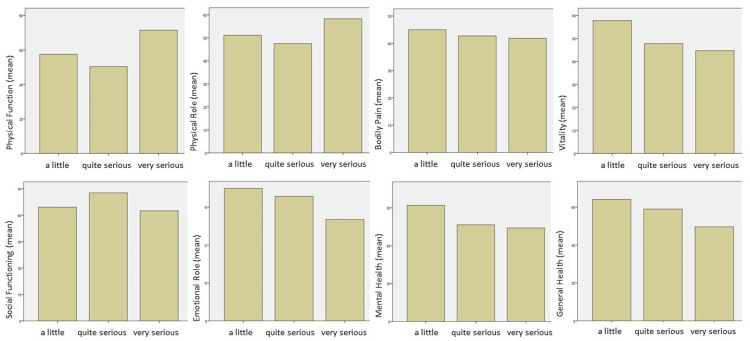
SF-36 mean scores per category based on the patient’s answers to the question: “How serious do you feel your health condition is?” SF-36, 36-Item Short Form Survey Instrument

**Figure 2:**
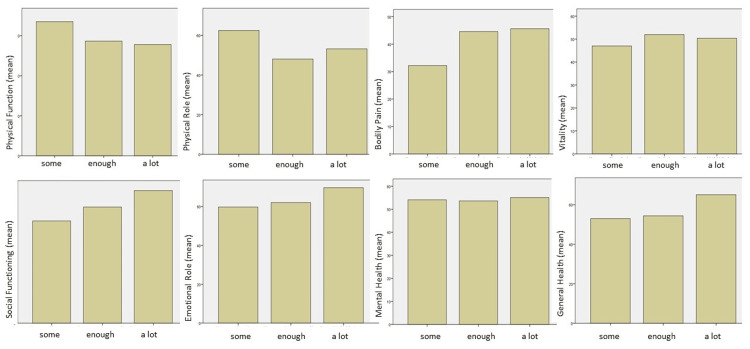
SF-36 mean scores per category based on the patient’s answers to the question: “To what extent do you feel informed about your health condition?” SF-36, 36-Item Short Form Survey Instrument

The physical functioning scale displayed significant differences based on the patients’ answers regarding their thoughts on the seriousness of their condition, as patients dealing with a more serious health condition scored higher results in physical functioning (Kruskal-Wallis p-value=0.015, Dunn’s post-hoc analysis p-value=0.006 between quite and very serious). The extent of information the patients stated knew about their condition did not affect significantly the SF-36 results. Interestingly, except for the physical functioning and role scales, even though patients with more serious health conditions reported worse scores in the other sections of their lives (with no statistical significance), the level of information seems to increase the quality of life in all categories (Figures 1, 2).

**Figure 3:**
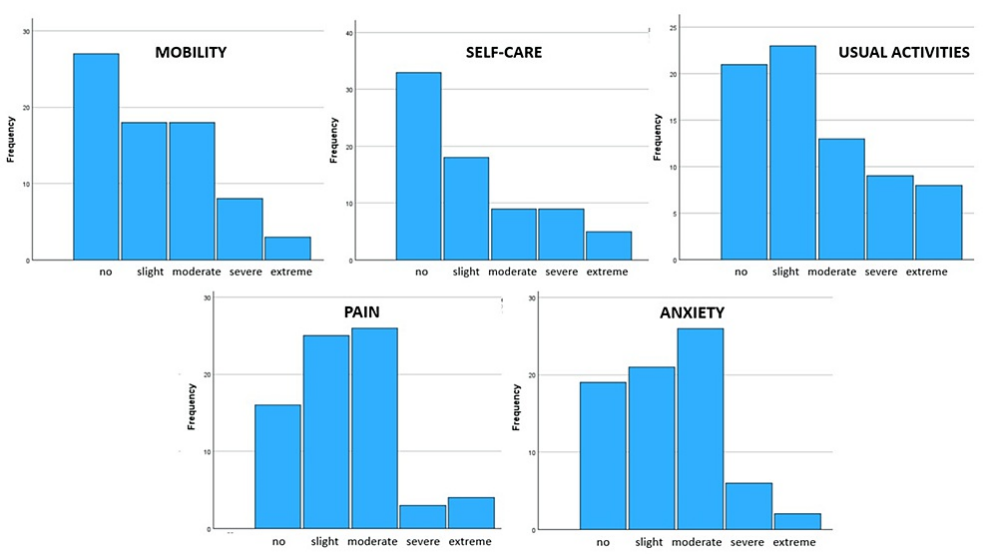
EQ-5D-5L questionnaire results per category

Regarding the EQ-5D-5L questionnaire, more than half of the participants noted that they presented no or slight problems, whereas less than 10% of total patients reported extreme problems (Figure 3). 

**Figure 4:**
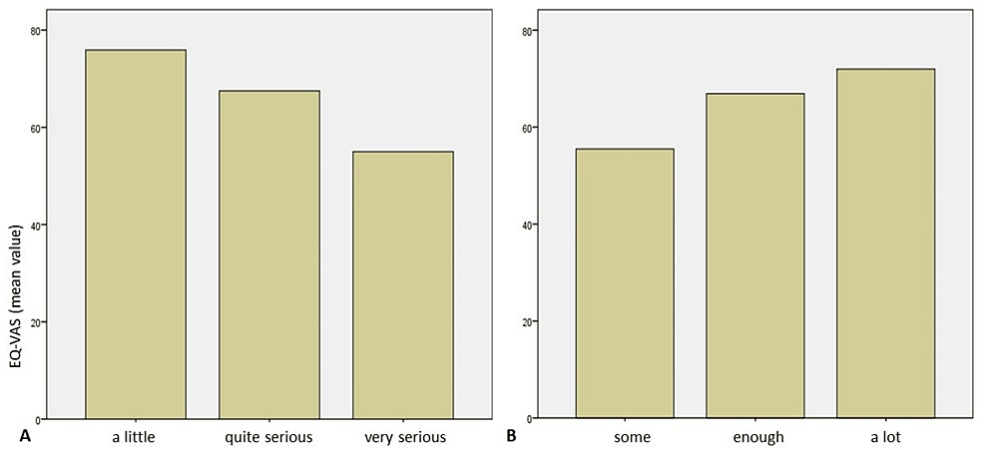
EQ-VAS mean scores based on the answers to the questions: “How serious do you feel your health condition is?” (A) and “To what extent do you feel informed about your health condition?” (B) VAS, visual analog scale

EQ-VAS mean score was found to be 67.42 (ranging between 20 and 95, with an SD of 19.85) and did not present any significant differences between genders, education level, and living environment, as noted in Tables 1, 2. EQ-VAS score was higher for younger patients (Kruskal-Wallis p-value=0.009, Dunn’s post-hoc analysis p-value=0.006 between patients <39 and 40-59 years old and 0.004 between patients <39 and >60 years old), while differences between older patient groups were insignificant. As far as the additional questions asked are concerned, the seriousness of their health condition affected significantly the quality of life (Kruskal-Wallis p-value=0.004, Dunn’s post-hoc analysis p-value=0.001 between a little and a lot serious and p-value=0.046 between quite and very serious), with decreasing scores in more serious conditions. Finally, even though the extent of information patients have regarding their condition did not seem to significantly differentiate the EQ-VAS scores (Kruskal-Wallis p-value=0.054), there is a clear trend that the more information the patients have, the better they score (Figure 4).

## Discussion

Quality of life is a general and abstract concept that represents the well-being of a person or a population and many efforts have been made to define and measure it using objective metrics. It has been thought that quality of life represents the level of satisfaction and contentment one gets in everyday endeavors, based on cognitive and emotional factors [6-9]. Coefficients that impact the quality of life have been studied in the literature and they mainly include social, such as environmental issues, availability of public services, and crime; and personal factors, such as income, housing, education status, employment, and work environment, personal relationships, sense of security, autonomy, and personal health status. The World Health Organization defines quality of life as a subjective evaluation of a person’s perception of reality based on their culture and value system [1]. In order to describe health-related quality of life, several measuring tools have been used, usually including questionnaires of self-assessment. 

In the present study, the aim was to assess the quality of life reported by neurosurgical patients, taking into account the perceived seriousness of their health condition and the level of information they have about their disease. The SF-36 and the EQ-5D questionnaires were used to achieve this goal, and the results were analyzed in depth and compared to each individual’s answer regarding the aforementioned parameters. It should be noted that our study has several limitations, including the relatively small sample size, the self-reported data, and the inability to categorize patients based on the specifics of their disease. These limitations affect our ability to accurately represent the broader population, and further research is required.

Our results did not demonstrate a statistically significant difference between genders, while age groups had different results on general health perception and EQ-VAS scores (patients younger than 40 years old scored significantly better). The impact of gender on the quality of life has been thoroughly studied in the bibliography, with several controversies, as other studies performed on the Greek population concluded that female patients reported lower scores than male patients [4,5,10]. Ungureanu et al. (2018) studied the quality of life of patients after spinal fusion and showed that even though the reported quality of life improved in both genders postoperatively, disability decreased significantly more in males [10]. Regarding age, our results are in agreement with the conclusions of Gil et al. (2003) who studied patients with anterior skull base tumors, as older patients had worse scores than younger ones [11]. On the contrary, another study that included vestibular schwannoma patients concluded that older patients had a better quality of life postoperatively, even though younger patients were more likely to change their profession [2]. Improved quality of life was also noted in patients older than 60 years old by Lytrivi M (2017) and this difference was attributed to the advantages of retirement in the perioperative period [4]. 

​​​In the present study, the patient’s living environment and education level affected significantly the reported physical functioning. More specifically, patients living in a rural environment and secondary education graduates reported higher functioning, probably due to the better physical shape the rural population usually displays; in fact, higher education graduates had the worst scores in regard to physical functioning. Physical functioning was also affected by the perceived seriousness of the patient’s health condition, as patients dealing with a more serious health condition scored higher results. Even though vitality did not seem to differ among our groups, another study showed better scores regarding the vitality of patients living in an urban environment, as opposed to those living in rural regions [4]. Zyoud et al. studied the quality of life among patients on hemodialysis based on the EQ-5D questionnaire; in this setting, the authors found that male patients, patients of higher education, and those living in rural areas scored the highest scores. However, the nature of the health condition studied should be taken into account, as it may cause different inconveniences during everyday tasks [12]. 

Based on our results on the patient’s self-reported seriousness of their health condition, 24.4% of the participants noted that their health condition is very serious and 40.5% that it is quite serious. Neurosurgical patients are a special subset of patients requiring postoperative care and potentially chronic treatment. Thus, the correlation between the patients’ perceived severity of their health condition and their quality of life, as described by the EQ-5D questionnaire, is reasonable. In fact, as it is presented in Figure 4, the more serious the patient’s health condition was, the lower the reported EQ-VAS score (Figure 4). On the other hand, most of the patients (86.4%) noted that they had at least enough information regarding their disease; even though the observed differences were not statistically significant, in most questions asked, the level of information the patient had regarding their health condition seemed to increase their reported quality of life (Figures 2, 4).

## Conclusions

The quality of life reported by neurosurgical patients is affected mainly by the patient’s age, while the living environment and education level have a clear impact on their reported physical role. Even though the perceived severity of the disease has a significant impact on the overall reported quality of life, well-informed patients seem to display better scores. Most parameters that influence the patient’s quality of life are fixed; thus, healthcare professionals should prioritize providing comprehensive information regarding the patient's health condition to improve the patient’s living standards.
